# Quality Control and Traceability Framework of Electrochromic Materials Based on Block Chain from the Perspective of Practical Application

**DOI:** 10.1155/2022/7376168

**Published:** 2022-08-03

**Authors:** Qiyuan Wang, Jingzhi Li, Muhong Zheng, Xie Ma, Bin Wang

**Affiliations:** ^1^Ningbo University of Finance and Economics, Ningbo, China; ^2^Hefei University of Technology, Hefei, China; ^3^Bank of China Anhui Branch, Hefei, China

## Abstract

In recent years, the research on electrochemical devices, especially the promising electrochromic material, is gradually in a wide range of application. But there is few corresponding research about specific industrial manufacturing. The paper starts from the structure of small and medium-sized intelligent factories and designs a system framework optimized by blockchain technology. It connects the electrochromic material quality control module with the product traceability module through a blockchain-based server, which not only ensures the production quality but also effectively improves the product supply chain, which is significant for the development of the electrochromic field.

## 1. Background and Significance of Electrochromic Material Quality Control and Traceability Framework

Research on electrochromic materials has achieved initial results and shown broad prospects, but there is no literature on specific industrial application. Starting from the typical intelligent factory architecture of small and medium-sized enterprises, this framework standardizes data collected by heterogeneous devices through Supervisory Control And Data Acquisition (SCADA) [1–9]. At the same time, block chain technology can reduce the delay of network layer data transmission and improve the real-time corresponding speed of control. Based on this research, a system framework is designed, which uses digital twin technology to map virtual workshop through the physical workshop and collects data through the workshop production control system to detect the current state and predict the future state in real time, so as to improve product or service quality and realize comprehensive improvement of ecological chain quality [5–7].

### 1.1. Research Background of Electrochromic Material Quality Control and Traceability Framework

In the 1960s, Platt first proposed the concept of “electrochromic.” In 1969, S. K. Deb et al. first reported the Tungsten trioxide (WO_3_) electrochromic material, pointing out that WO_3_ can be rapidly switched under different electrochemical conditions. In the positive voltages (Oxidation), WO_3_ film is transparent; while it acquires a deep blue color under the negative voltages (reduced) [10–15].

**Figure 1:**
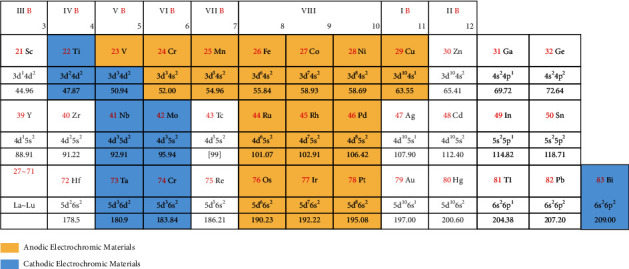
Most inorganic electrochromic materials are transition metal oxides.

Since then, electrochromic materials attracts wide attention from scientific researchers [16–20]. In the in-depth research, in the 1970s, scientists successively discovered that transition metal oxides such as MoO_3_, TiO_2_, IrO, and NiO also have electrochromic properties, and the relative development is also increasing. The inorganic electrochromic materials discovered so far are shown in Figure 1.

In summary, among the inorganic materials, materials that can be colored under cation insertion is “cathodic” EC material, or an “anodic” EC material (coloration under anion insertion), while the V_2_O_5_ is Amphoteric EC material [21–25].

Figure 1 in the periodic table of elements reports that most EC material belongs to transition metal elements so far. Compared with thermochromic materials, EC materials currently have unique advantages in the application of smart windows due to their great optical properties, fast switching response, high coloration efficiency, and good cycling stability. While inorganic electrochromic materials have excellent stability, making it easier to meet the needs of outdoor applications in the fields of displays, energy storage devices, and military infrared stealth [24, 25].

As the earliest EC material studied, WO_3_ has good reversibility, long service life, low cost, good coloration efficiency, and switching time, there is a fairly mature industrial system at present. Simultaneously, in the era of Industry 4.0 dominated by intelligent manufacturing, electrochromic materials can also play a great role in it. As an important functional film material, WO_3_ film has a wide range of application prospects in business, life, and national defense. In the era of industry 4.0 dominated by intelligent manufacturing, electrochromic materials can also be involved.

The chemical effect and amplification effect generated by the integration innovation of traditional manufacturing enterprises with the Internet of Things, information technology, automation technology, big data, and other technologies constantly promote the transformation of R&D, design, production and manufacturing, and marketing service mode. As for the traditional quality function, due to the gradual intelligence and digitalization of production, logistics, and other links, blockchain is a consensus mechanism established by any node in the entire network in a trustless environment, without worrying about data tampering, so as to realize point-to-point transaction, coordination, and collaboration. Its characteristics of decentralization, distributed shared ledger, trusted transaction and nonrepudiation are suitable for the field of product traceability. Blockchain technology, Internet of Things technology, artificial intelligence, digital twin technology, big data, and other technologies to strengthen information management services, improve the controllability of the production process, reduce human intervention, reasonable arrangement of production plans, and to maximize the benefits of enterprises.

In recent years, product traceability has focused on statistical quality management and around ISO 9000. In 2018, Lee June Hyuck et al. designed a CPPS architecture framework for quality prediction and operational control in metal casting production. In 2019, Maciel Peixoto et al. used performance evaluation planning to carry out a multidimensional forecasting analysis and solve the impact of multidimensional factors on each response variable through performance evaluation planning. In 2020, Chen Gaige et al. applied virtual reality mapping and fusion, digital twin, big data drive, virtualization, edge computing, and cloud service technologies to manufacturing systems and developed a conceptual framework for CPS based discrete manufacturing intelligent factory paradigm [5, 14, 15].

### 1.2. Research Significance of Electrochromic Material Quality Control and Traceability Framework

At present, electrochromic devices (ECDs) are widely used in smart windows, sensors, energy-saving buildings, and other fields, but their optical modulation amplitude, switching time, color change types, and other properties still have room for improvement. We wish that there is a way to further explore the properties and practical value of materials, optimize the production process, and make it contribute to the development of society [3, 25].

In the field of product traceability, there are no relevant literature studies on the intelligent industrial chain specifically using blockchain technology. Using this technology can reduce the delay of data transmission at the network layer and improve the real-time control, which has academic and application value.

### 1.3. Research Characteristics of Electrochromic Material Quality Control and Traceability Framework

#### 1.3.1. Research Characteristics of Quality Control and Traceability Framework

*(1) The Overall Structure is Novel*. Based on blockchain technology, big data, Internet of Things technology, digital twin technology, automation technology, etc., the architecture of an intelligent factory (intelligent workshop) is studied.

*(2) Research on Traceability of Industrial Chain Based on Block Chain Technology*. Based on the upstream and downstream quality data of the industrial chain, the accuracy of products and components can be reasonably determined through spectrum analysis, wavelet multiresolution analysis, error tracing and decomposition, etc., so as to build an industrial chain quality management model, optimize product design, optimize MES and ERP, and improve the performance of the industrial chain as a whole.

#### 1.3.2. Characteristics of Electrochromic Material Manufacturing Method

**Figure 2:**
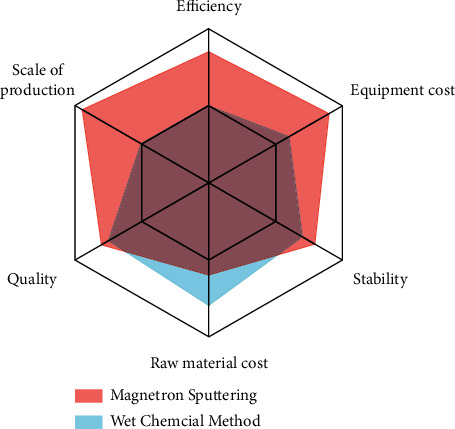
Comparison between the traditional preparation method and magnetron sputtering method.

In the past, people used to prepare thin films with wet chemical methods. Due to the complex reaction process and the difficulties in regulating the process, it was hard for EC to separate from the laboratory and realize industrial production. Now, the maturity of magnetron sputtering technology makes it possible to mass-produce EC. The latter is an emerging thin film preparation technology. Although the magnetron sputtering equipment is quite expensive. However, compared with the traditional wet chemical preparation, its production efficiency and production scale are far superior to the former, and the production process of magnetron sputtering is more stable and reliable, the film product is more uniform. The picture data are obtained by quantification sorting [1, 2]. A comparison between the traditional preparation method and magnetron sputtering method is shown in Figure 2.

## 2. Introduction of Electrochromic Material Quality Control and Traceability System Framework

At present, the application direction of most blockchain is mainly the transaction of digital currency. However, due to the inherent immutable and unerasable characteristics of blockchain technology, it can be used in the information storage of the whole chain of the supply chain to form the transmission of credit value. The traceability module of the system uses blockchain technology. Due to its decentralized and distributed ledger characteristics, the product traceability method of the system has the characteristics of safety, reliability, nontampering, and traceability. Workshop production control system mainly through a real-time batch sampling of products to analyze the secondary product rate, control the production mode of products, to ensure the quality of products.

### 2.1. Digital Model of Electrochromic Material Quality Control and Traceability System Framework

This digital model mainly uses digital twin technology, block chain technology, big data, Internet of Things technology, automation technology, etc. Digital twin workshop is the main application place of digital twin technology at present. Digital twin workshop refers to the bidirectional mapping and real-time interaction between the virtual workshop and physical workshop. Physical workshop virtual workshop and workshop production control system can be integrated to form a new workshop control mode [9, 11–13]. Physical workshop and specific workshop for production and manufacturing, the main components for a variety of production, testing equipment, part of the management personnel, is the physical basis. A virtual workshop is a virtual mapping of a physical workshop. It is a simulation object generated based on digital twin technology. It can be used for interaction with physical workshop, data acquisition and prediction, and other functions.

**Figure 3:**
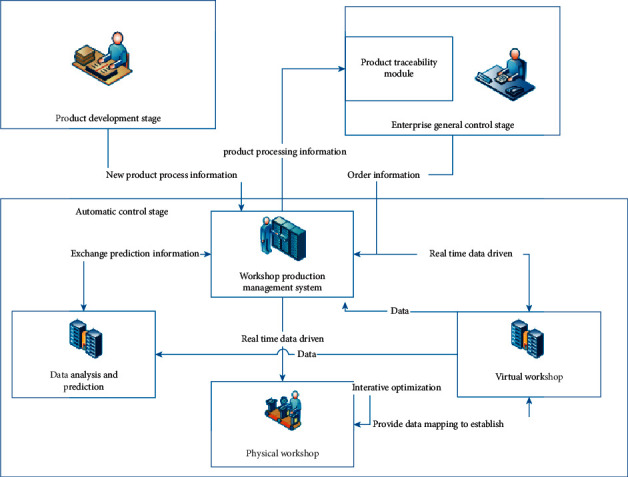
A digital model.

Data collected by heterogeneous devices is standardized by Supervisory Control And Data Acquisition (SCADA). A workshop production control system is a tool used by enterprises to manage production plans and product quality. It is an important bridge connecting physical workshop and virtual workshop.  Figure 3 is a digital model.

The data prediction in Figure 3 mainly relies on the Markov prediction model. This model can predict its future state by using the current state and changing trend. It is mainly used to analyze the future variation trend of discrete stochastic processes. The application of the Markov method is based on the premise that the whole process of the thing to be predicted conforms to the Markov process, which is a random process without an aftereffect. In other words, when the state of the process at a certain moment is known, the probability of the state of the process at the next moment is only related to the state of the process at a certain moment and has nothing to do with the state before a certain moment. Data prediction can be used to collect data and change trend through the detection equipment of the workshop production control system to predict the future state, which plays an important role in the quality control system [10].

### 2.2. Workshop Production Control System Framework

The specific implementation method of the production and secondary detection module is to use a field emission scanning electron microscope (FESEM) and X-ray photoelectron spectrometer (XPS) to detect all aspects of performance information of materials during initial production. If the quality of materials is failed, the batch of products will be stopped in order to prevent defective products from entering the market, this batch of products will be recycled to ensure product quality.

After the device is assembled, the ultraviolet-visible-near-infrared spectrophotometer (UV) is used as the final electrochromic property characterization instrument. If the properties of the ECDs product are lower than the expected while is little impact on the basic service, the product is classified as a defective product and be sold at low prices through defective sales channels because of nonremovability after assembling into the device. Of course, products that have a serious impact on performance are directly discarded. The adoption of production and secondary inspection modules greatly ensures product quality and significantly reduces the possibility of defective products entering the market.

**Figure 4:**
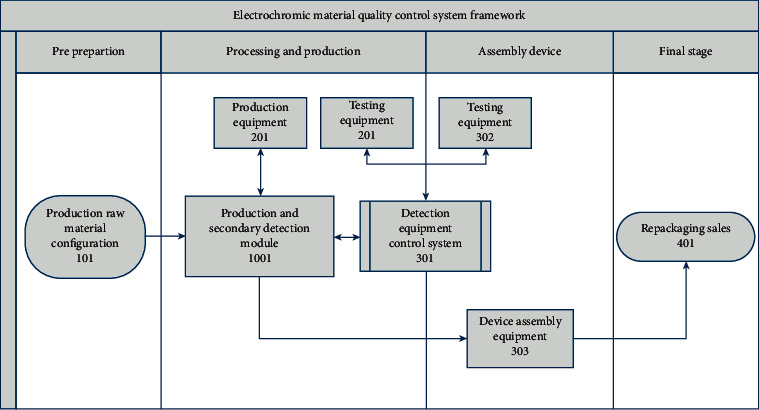
Framework of electrochromic material quality control system.

In this paper, We choose the production process of WO_3_-x, which is widely used in common inorganic electrochromic materials, to prepare EC thin films on substrate glass by magnetron sputtering.  Figure 4 is the framework of the electrochromic material quality control system. The modules in Figure 4 are explained as follows:

  101: production raw materials configuration are equipped with high-purity W target as the main raw material, FTO (Tin oxide doped with fluorine) conductive glass as the substrates.
  1001: production and secondary inspection module manage all production and monitoring equipment. This module requires a magnetron sputtering vacuum coated machine as the main production device. FESEM and XPS are used as sampling morphology observation and control equipment during the processing stage, and UV is used as the final electrochromic property characterization instrument in the final assembling device stage.
  201: indicates that in the ultrahigh vacuum dc magnetron sputtering machine, the desired product is prepared by the impact of high-energy particles and the sputtering deposition of substances, using a high-purity W target (99.99%) as the target raw material, (99.99%) Argon gas is used for sputtering (99.99%) oxygen is used as the reactive gas, and the thin film is deposited on the conductive glass of the FTO substrates
  202: topography control, in the magnetron sputtering, the EC thin film material is prepared, and before it assembles into the device, the product is sampled and monitored at a ratio of 100 : 1, through XRD, XPS, and SEM. The topography, phase, and element valence state are characterized to ensure the preliminary reliability of the product.
  302: assemble the final device, in the 301 step, we use magnetron sputtering to synthesis the EC layer (EC) of the device, there still needs the electrolyte layer (IC) and the ion storage layer (CE&IS). Considering to meet the needs of mass production, NiO can be used as the CE layer to form a complementary ECDs, and use solid electrolyte to ensure the safety of the product.
  303: After assembling into the device, monitor the EC properties of the product, and use a spectrophotometer to test the properties of the ECDs through sampling inspection. The common EC properties include optical modulation amplitude, coloration efficiency, cycling stability, switching time, and optical memory effect. And, the first three properties is more vital for the mass production. Through the spectrum, we observe the coloration of the product to ensure that the product has a high optical modulation amplitude. If the amplitude reaches the expect, then we take the next step to test the coloration efficiency to evaluate the energy consumption of the product. The third we test cycling stability, which determined the service life of the product. As the test data reach the standard, the detection is completed and the product is sold in subpackages.
  404: it is repackaging sales, the sales channels for standard and defective products are different, but they all use the EC material traceability system to standardize data collection and upload them to the system in real time for traceability.

#### 2.2.1. Innovation of Workshop Production Control System Framework

*(1) The Advantages of Magnetron Sputtering Vacuum Coating Method*. Under the influence of the magnetic field force, the energy utilization rate of the high-energy particles and the ionization rate of the working gas are incredibly increased, so that the air pressure in the equipment can be greatly reduced under the premise of satisfying the sputtering conditions. Simultaneously, the electrons whose energy is exhausted would not release too much heat energy when they impact the substrate, hence, it greatly reduces the damage to the substrate glass;

Furthermore, sputtering at a lower gas pressure can not only reduce the scattering of sputtered particles by gas molecules, improve the deposition efficiency, but also increase the bonding force between the substrate and the film to avoid the film layer falling off. High-energy particles are bound near the target due to the effect of the magnetic field force, which is not only conducive to the bombardment of particles on the target to sputtering efficiency and increases deposition rate indirectly.

**Figure 5:**
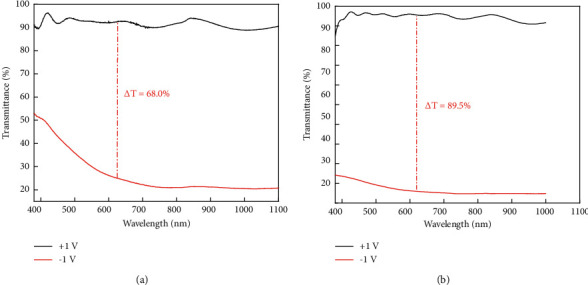
Comparison of coloring efficiency between wet chemical method and magnetron sputtering method.

We prepared the same thickness WO_3_ films in different ways (the thin film is bleaching state under positive voltage, and in a coloring state under negative voltage). As shown in Figure 5, the left is using the classical wet chemical method and spin coating, and the right is taking magnetron sputtering method, both of them are experimented ten times. △T indicates the difference of optical transmittance in the bleaching and coloring state at 633 nm. It clearly shows that the product coloring obtained by the magnetron sputtering method The efficiency is higher than the wet chemical method.

*(2) Advantages of the Control System*. Based on multisensor data fusion, the Markov prediction model is adopted to store the calculation results into the database, which reduces the amount of cloud computing and defective rate, as well as the delay of a large amount of data access and calculation. The database considers ERP, MES data format compatibility, and ease of transformation. Through the method of secondary detection, the morphology detection is carried out before the device is assembled, and the final electrochromic performance characterization detection is carried out after the device is assembled, which greatly improves the quality of the product and avoids the defective products entering the market.

### 2.3. Traceability System Framework Based on Block Chain

The system uses Solidity, the current official recommended language for developing smart contracts. The blockchain platform of the system framework uses Ethereum, and the execution environment of smart contracts is the sandbox environment using Ethereum virtual machines as an isolated environment. It avoids the situation that the smart contract is affected by the attack, thus improving the overall robustness of the traceability system.

The traceability system framework is centered on the blockchain platform, and the production and secondary inspection module, supply chain information module, login module, and franchisee module are important components. Including production and secondary detection module will bear record product feature information and the task of production information, a detailed record of each batch of product information upload block chain platform and put a unique identification code, users can scan the code learned that their production information, and if the hidden defects of the product after the user told enterprise, Enterprises can retrieve this batch of products by querying the information in the blockchain platform to improve their reputation and product quality. The supply chain information module undertakes the task of recording the information of the whole chain process.

**Figure 6:**
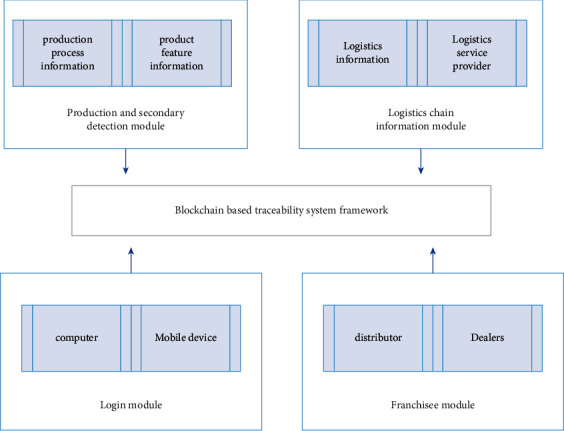
Product traceability module.

The characteristics of the block chain that cannot be tampered with and deleted reliably ensure the retention of the historical records of the whole chain and improve the security of the logistics chain. The login module allows users to log into the blockchain platform of the system framework to query relevant information or contact customer service for feedback. Franchisees module can let franchisees in different interface different permissions to enter this platform, for example, consumers can only through the unique identification code query log in the product information, and logistics information, but the joining trader can through the enterprise special account access to cooperate with our company information in a series of products, logistics information, etc [4, 8, 18–23].  Figure 6 is the product traceability module.

### 2.4. General Introduction of Electrochromic Material Quality Control and Traceability System Framework

**Figure 7:**
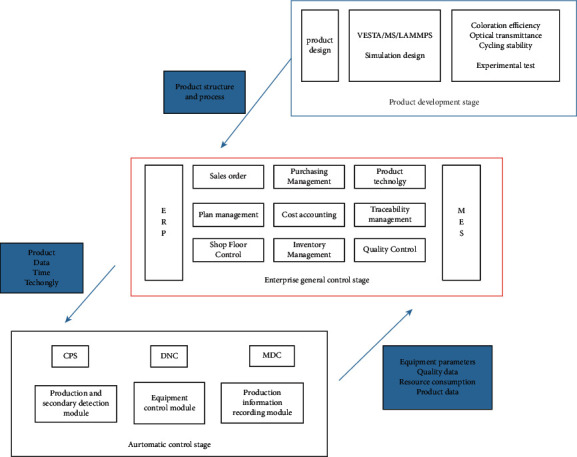
Electrochromic material quality control and traceability system framework.

The overall framework of the electrochromic material quality control and traceability system is shown in Figure 7. This framework starts from the product development stage, which consists of two parts. Simulation design is carried out first, and experimental test is carried out later. In this stage, the product structure and process are transferred to the next stage. In the overall control stage, MES or ERP is adopted to improve the enterprise structure and optimize the system to improve the quality of products and services. The enterprise monitors and controls the production of products through the quality control module and digital workshop control module, and transmits the quantity, type, and time of products required by the order to the next stage. In the automatic control stage, digital twin technology, CPS, DNC, and MDC technology are used to improve the system to achieve the purpose of improving productivity and robustness. Production and secondary inspection module can effectively improve product quality to avoid defective products entering the market. The equipment control module can reduce equipment loss and improve the utilization rate. The product information recording module can record the product information and upload it to the blockchain platform in time, which is conducive to product traceability.

## 3. Conclusions

After checking, the industrial production of electrochromic materials combined with block chain technology, big data, digital twin technology, information technology, automation technology, and network technology has not been reported in the relevant literature. The existing electrochromic materials have a narrow application range and few practical applications, which have broad development space and strong market potential. An innovative mass production method is proposed in this paper. The main production method is the advanced magnetron sputtering vacuum coating method. The production and secondary inspection module are used to detect the product quality twice in the production and processing stage and the device packaging stage, which greatly reduces the defective rate.

This system through the quality control system based on block chain technology framework for testing implement quality control, to prevent the imperfect product into the market, through the block chain platform to product research and development, enterprise general control phase, automatic production stage together, convenience for the user login module to read of production information and realize the product traceability. The product quality control is also convenient for consumers to trace the product, easy to operate.

## Data Availability

The dataset analyzed during the current study is available from the corresponding author on reasonable request.
